# *Lactobacillus plantarum* RS-09 Induces M1-Type Macrophage Immunity Against *Salmonella* Typhimurium Challenge via the TLR2/NF-κB Signalling Pathway

**DOI:** 10.3389/fphar.2022.832245

**Published:** 2022-03-07

**Authors:** Chenpei Zhao, Huan Chen, Hao Liang, Xiaoyu Zhao, Wenli Tang, Maolian Wei, Youzhi Li, Jianlong Zhang, Xin Yu, Guozhong Chen, Hongwei Zhu, Linlin Jiang, Xingxiao Zhang

**Affiliations:** ^1^ School of Life Sciences, Ludong University, Yantai, China; ^2^ Department of Microbiology, School of Public Health, Cheeloo College of Medicine, Shandong University, Jinan, China; ^3^ Shandong Provincial Key Laboratory of Quality Safty Monitoring and Risk Assessment for Animal Products, Jinan, China; ^4^ Yantai Key Laboratory of Animal Pathogenetic Microbiology and Immunology, Yantai, China; ^5^ Shandong Aquaculture Environmental Control Engineering Laboratory, Yantai, China

**Keywords:** *Lactobacillus plantarum*, *Salmonella typhimurium*, macrophage, macrophage polarization, M1 macrophage, TLR2

## Abstract

*Lactobacillus plantarum* can interact with macrophages against bacterial enteropathy due to its potential ability to modulate macrophage polarization. However, this mechanism is not completely understood. TLR2 can recognize microbial components and trigger macrophage cytokine responses to different gram-positive strains. The aim of this study was to investigate whether probiotic *Lactobacillus plantarum* RS-09 can induce macrophage polarization against *Salmonella* Typhimurium infection via TLR2 signalling. BALB/c mice were preadministered RS-09 continuously for 7 days and then infected with *Salmonella* Typhimurium ATCC14028. Mouse RAW264.7 mononuclear macrophages were stimulated with RS-09 and coincubated with ATCC14028 or PBS controls. The results of the *in vivo* study indicated that RS-09 could relieve *S*. Typhimurium-induced splenomegaly, body weight loss and death rate. RS-09 also limited the colonization and translocation of *S*. Typhimurium in the gastrointestinal tract and thereby protected against infection. We also observed that RS-09 upregulated the production of M1 macrophage characteristics (e.g., CD11c and IL-6) against *S*. Typhimurium. Furthermore, RS-09 induced the expression of TLR2 in macrophages. In an *in vitro* study, treatment of RAW264.7 cells with RS-09 either concurrently with or before *S*. Typhimurium challenge enhanced the secretion of Reactive oxygen species and Nitric oxide. This effect was related to TLR2 and NF-κB activation. Based on these findings, *Lactobacillus plantarum* RS-09 was shown to modulate M1 macrophage polarization and induce TLR2-linked NF-κB signalling activity in the innate immune response to *S*. Typhimurium infection.

## Introduction

*Salmonella enterica* Typhimurium (*S*. Typhimurium) is an important foodborne pathogen that causes enteric diseases in humans and animals (Dougan et al., 2011; Broz et al., 2012; Riddle and Porter, 2012). The innate immune system is a primary defence mechanism against pathogen infection involving mononuclear phagocytes, such as macrophages, neutrophils, dendritic cells, and monocytes. Macrophages play an essential role in the host response to *S.* Typhimurium infection. Accumulated evidence has shown that *S*. Typhimurium can survive and replicate in macrophages to evade the host response. *S*. Typhimurium can influence the macrophage phenotype and polarize M1 to M2-like macrophages, thus escaping the immune system (Brumell et al., 2001; Antunes et al., 2011). Therefore, the role of the M1 phenotype is in favour of antimicrobial activity in controlling infection.

Increasing evidence has demonstrated that the immune response of macrophages is initiated after pathogen recognition through Toll-like receptors (TLRs), including phagocytosis, pro- and anti-inflammatory cytokine release, oxidative burst activation and M1/M2 macrophage polarization (Atri et al., 2018). Toll-like receptor 2 (TLR2), a member of the Toll-like receptor family expressed in macrophages, is critical for triggering innate immune responses by recognizing microbe-associated molecular patterns (MAMPs, bacterial cell wall components) (Shida et al., 2009; Kaji et al., 2010; Rocha-Ramirez et al., 2020). Furthermore, the TLR2 signalling pathway plays a prominent role in modulating macrophage differentiation to either M1 or M2 after pathogenic bacterial infection. Previous experimental data showed that bacteria can induce M1 macrophages to produce a large amount of proinflammatory mediators, including Tumor necrosis factor-α (TNF-α), Interleukin-1(IL-1), and Nitric oxide (NO), via the TLR2 signalling pathway during the early and acute stages of bacterial infection (Khan et al., 2015). Even though pathogens are recognized by macrophages through TLR2, pathogens may utilize TLR2 as part of their survival mechanism. When the infection persists, macrophages polarize to the M2 phenotype to survive in the hostile environment created by macrophages (Yoshida et al., 2009; Lim et al., 2016; Kewcharoenwong et al., 2018). This makes TLR2 an intriguing molecule to study to understand its role in the regulation of the immune response.

Probiotics have been shown to have a wide range of biological activities, including immune regulation (Sánchez et al., 2017), improvement in the intestinal environment (Collins and Gibson, 1999), and direct antimicrobial activity against pathogens through direct contact with immune cells (Dunne et al., 1999; Corr et al., 2007). Probiotics also have MAMPs that can be recognized by TLR2 on macrophages to trigger the immune response, such as peptidoglycan, lipoteichoic acids and the cell wall (Takeda and Akira, 2004). For instance, *Bacillus amyloliquefaciens* (Ba) can facilitate polarization of M1 macrophages and increase the expression of Interleukin-1β (IL-1β), Inducible Nitric Oxide synthase (iNOS), TNF-α and Interleukin-6 (IL-6) (Ji et al., 2013). *Lactobacillus rhamnosus R0011* (LrF) induced Interleukin-10 (IL-10) and IL-1β production and the expression of CD206, markers of immunoregulatory M2 macrophage polarization (Jeffrey et al., 2020). *Lactobacillus rhamnosus GG* (LGG) induced M1 macrophage polarization and M1-related cytokine expression against pathogen invasion (Baikui Wang et al., 2020). Thus, when the body encounters microbes or their components, the immune response following MAMP-TLR2 recognition is diversified and dependent on the biochemical characteristics of both the bacterial and host cells.

Among probiotic strains, *Lactobacillus plantarum* (*L. plantarum*) is known for its potential probiotic properties, such as human safety, immunoregulatory activity and anti-inflammatory effects. Accumulated evidence has shown that *L. plantarum* can enhance host immune responses mainly by modulating cytokine production and manipulating macrophage phenotypes to exert immunoregulatory effects (Wang et al., 2008; Sichetti et al., 2018; Kim et al., 2020). However, whether *L. plantarum* activates the macrophage-polarizing effect against *Salmonella* infections through TLR2 signalling is not well established. To address this question, we investigated the protective effect of *L. plantarum* RS-09 challenged with pathogenic *S*. Typhimurium on the polarization of macrophages and explored the involved signalling pathways.

## Materials and Methods

### Strains and Growth Conditions

The probiotic *L. plantarum* RS-09 was isolated from fermented apple and kept at the China General Microbiological Culture Collection Center (CGMCC No. 17118). *S*. Typhimurium ATCC 14028 (Sty14028) was preserved in our laboratory. From frozen stocks (−80 °C), *L. plantarum* RS-09 was suspended in Man-Rogosa-Sharpe liquid medium (MRS broth; Solarbio, Beijing, China), cultured anaerobically at 37 °C for 48 h in fresh MRS broth (three–four OD at 600 nm), and harvested by centrifuging at 4,000×g for 15 min. *S*. Typhimurium (Sty14028) was grown in Luria-Bertani medium (LB broth; Solarbio, Beijing, China), cultured at 37°C for 24 h (1–1.5 OD at 600 nm), and harvested by centrifugation at 6,000×g for 20 min.

For the *in vitro* study, after three washes with phosphate-buffered saline (PBS, pH = 7.2; BBI, Shanghai, China), the bacteria were resuspended in DMEM (antibiotics-free; Gibco, CA, United States) for preincubation or infection. For the *in vivo* study, the bacteria were administered orally to mice in 1 ml of PBS.

### Cell Culture

The leukaemia cells of the mouse mononuclear macrophage RAW264.7 cell line were cultured in DMEM (Gibco, CA, United States) supplemented with 10% foetal bovine serum (fetal bovine serum, FBS; Gibco, CA, United States) in a humidified incubator at 37°C and 5% CO_2_. RAW264.7 cells were subcultured every 2-3 days.

### Animals

Female SPF-grade BALB/c mice (4-5 weeks old) were obtained from HFK Bioscience Inc., (Beijing, China) and maintained in the Experimental Animal Center of Ludong University. All animal experiments were conducted according to the guidelines and approval of the Laboratory Animal Welfare and Ethics Committee of Ludong University. All mice were raised in an air-conditioned and specific pathogen-free environment (60% RH, 22°C, light exposure from 8:00 to 21:00) with free access to sterilized food and water.

### Bacterial Infection Assay in Mice

**FIGURE 1:**
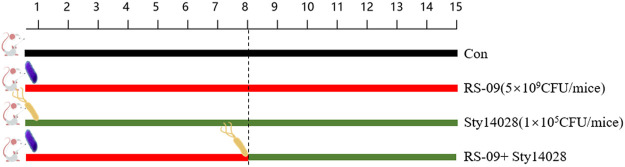
Experimental design of the mouse study.

Figure 1 shows the experimental design. Twenty-four mice were randomly divided into four groups (six mice per group): normal control group treated with PBS alone, control group treated with Sty14028 alone, one group treated with *L. plantarum* RS-09, and one group treated with *L. plantarum* RS-09 with *S. typhimurium* 14,028. The RS-09 group and RS-09 with Sty14028 group received an intragastric administration of 200 μL of a suspension containing 5 × 10^9^ CFU/mouse of live RS-09 once daily for 7 consecutive days *via* a feeding needle. On the 8th day of RS-09 in the Sty14028 group, mice were infected with a 200 μL median lethal dose of S. typhimurium 14,028 (1 × 10^5^ CFU/mouse). All animal weights and survival rates were monitored daily, and mice were euthanized 8 days post-challenge.

### Determination of the Spleen and Liver Index

The spleen and liver were harvested from the mice in each group. The whole spleen and liver were weighed immediately, and the organ index was expressed as the ratio of the organ weight to body weight (mg/g) (Habil et al., 2011).

### *S*. Typhimurium Colonization in the Spleen, Liver and Intestinal Segments

The spleen and liver were cut and homogenized using a tissue tearer (TIANGEN, Beijing, China) with 5 ml of PBS. The contents of different intestinal segments were harvested in 5 ml PBS. Serial dilutions were plated onto XLT-4 agar (Hopebiol, Qingdao, China) and incubated at 37°C for 12 h, and the bacterial colonies were recorded as logarithm base 10 (log_10_ CFU/g).

### Blood Sampling

For each mouse, 1.0 ml of blood from the caudal vein was collected. The blood samples were divided into two parts. One was treated using sodium citrate to obtain anticoagulated whole blood to detect routine histopathology by an automatic blood cell analyser (Rayto RT-7600S, Shenzhen, China), and the other was stored at 4°C for 4 h to collect serum for cytokine measurements.

### Cytokine Quantification

Cytokines, including mouse IFN-γ, IL-6, and Interleukin-12 (IL-12), were quantified in serum using enzyme-linked immunosorbant assays (ELISA) following the manufacturer’s protocols (R&D Systems, MN, United States). ELISA for IL-10 (Cat. no 430603) using serum was also performed following the manufacturer’s protocols (BioLegend, CA, United States). All ELISAs were performed using 96-well high-binding ELISA plates in ELISA kits, and plates were read at a wavelength of 450 nm using a Synergy HTTR microplate reader (Bio-Tek Instrumentation, VT, United States).

### Flow Cytometry (FCM) Analysis of Macrophage Surface TLR2 Expression

To detect the cell surface expression of TLR2, cells were incubated with antibodies against mouse TLR2-FITC, CD11c-PE and CD206-APC for 30 min at room temperature, washed and analysed in a FACS Calibur flow cytometer (BD Biosciences, New York, United States).

### Macrophage Stimulation in Vitro

For macrophage polarization assays, RS-09 and Sty14028 were collected by centrifugation and washed three times with PBS. RAW264.7 cells were seeded into a 24-well culture plate at a concentration of 2 × 10^6^ per well. RS-09 was then applied to infect macrophages at an MOI of 20:1 (4 × 10^7^ CFU: 2 × 10^6^ RAW264.7) for 12 h (*n* = 3 for each group). RAW264.7 cells were then washed with PBS twice. Sty14028 was then applied to infect the macrophages at an MOI of 20:1 (4 × 10^7^ CFU: 2 × 10^6^ RAW264.7). After incubation for 1 h, the cells were washed three times and treated with 200 μL of gentamicin sulfate (100 μg/ml) for 1 h. RAW264.7 cells were then washed with PBS twice and incubated for 12 h with DMEM (including 20 μg/ml of gentamicin sulfate). The cells were harvested for the detection of macrophage phenotype, reactive oxygen species (ROS), and proteins with altered expression, and culture supernatants were simultaneously assayed for NO.

### Flow Cytometry (FCM) Analysis of Macrophage Polarization

CD11c and CD206 are markers of the M1 and M2 macrophage phenotypes, respectively. To analyse the activation effects of RS-09 on macrophages, treated RAW264.7 cells were incubated with fluorochrome-conjugated antibodies against mouse CD11c-PE and CD206-APC. CD206-APC was demonstrated by intracellular labelling. Cells were treated with FIX&PERM Kit MEDIUM A (MultiSciences, Hangzhou, China) for 15 min at room temperature and then incubated with FIX&PERM Kit MEDIUM B and CD206-APC for 30 min at room temperature followed by two washes in PBS. The percentages of CD11c^+^ cells (M1 macrophages) and CD206^+^ cells (M2 macrophages) were analysed on a FACS Calibur flow cytometer (BD Biosciences, New York, United States).

### Determination of Intracellular ROS Levels

The formation of intracellular ROS in bacteria-treated RAW264.7 cells was measured using an intracellular ROS detection kit (Beyotime, Shanghai, China) according to the manufacturer’s instructions. Briefly, cells were stained with dichlorodihydrofluorescein-diacetate (DCFH-DA, 10 μM) and incubated for 30 min at 37°C in a 5% CO_2_ humidified incubator. After washing twice with PBS, the cells were stained with 4,6-diamidino-2-phenylindole (DAPI) for 10 min at room temperature. RAW264.7 cells were then washed with PBS thrice. Cells were then immediately visualized through a fluorescence microscope and photographed (400×). The formation of intracellular ROS was also measured by a flow cytometer (FACS calibur; BD Biosciences, New York, United States) equipped with an argon laser, yielding a 488-nm primary emission line.

### Detection of NO

Cell supernatants were collected to determine the expression of NO by a nitrate/nitrite assay kit (Beyotime, Shanghai, China). Briefly, supernatants (60 μL) were collected and mixed with an equal volume of Griess reagent (1% sulfanilamide and 0.1% N-(1- naphthyl) ethylenediamine dihydrochloride in 5% phosphoric acid) at room temperature for 15 min. After mixing with Griess reagent Ⅰ and Griess reagent Ⅱ for 10 min at room temperature, supernatants were detected at 550 nm using a microplate reader (Biotek, VT, United States), and the actual concentration was calculated using a standard curve with serial dilutions of NaNO_2_ (Zhang et al., 2009).

### Western Blot

After treatment with bacteria, cells were washed with ice-cold PBS and lysed in RIPA lysis buffer containing 1 mMPMSF. With centrifugation at 12,000 rpm and 4°C for 20 min, the protein concentration was determined by the BCA protein quantitative method (Thermo, MA, United States). Equal amounts of protein (50 μg per lane) were separated by 12% sodium dodecyl sulfate–polyacrylamide gel electrophoresis (SDS–PAGE) and transferred to nitrocellulose membranes on ice. The membrane was blocked with 5% skimmed milk (Cell Signalling Technology, MA, United States) for 2 h at room temperature and incubated with iNOS (1:1,000, Cell Signalling Technology, MA, United States) and *β*-Actin (1:1000, Proteintech, Chicago, United States) antibodies at 4°C overnight. The protein was detected with horseradish peroxidase (HRP)-conjugated secondary antibody (1:2500, Cell Signalling Technology, Mass, United States) for 1 h aat room temperature. Finally, an ECL kit (SuperSignal™ West Pico PLUS, Thermo Scientific, MA, United States) was used to observe the protein bands using a gel imaging analytic system (Bio–Rad, CA, United States). The image grey value was analysed using AlphaEaseFC software (Alpha Innotech, CA, United States).

### Statistical Analysis

Graphs were analysed using GraphPad Prism Software version 6 (GraphPad Software, CA, United States) and are presented as the means ± SD of at least three independent experiments. Comparisons between pairs of data were assessed using 2-tailed Student’s t test and between multiple groups of data using one-way analysis of variance (ANOVA). Differences were considered statistically significant for *p* values <0.05.

## Results

### Effects of Pretreatment of Mice With *L. plantarum* RS-09 on *S*. Typhimurium Infection

**FIGURE 2:**
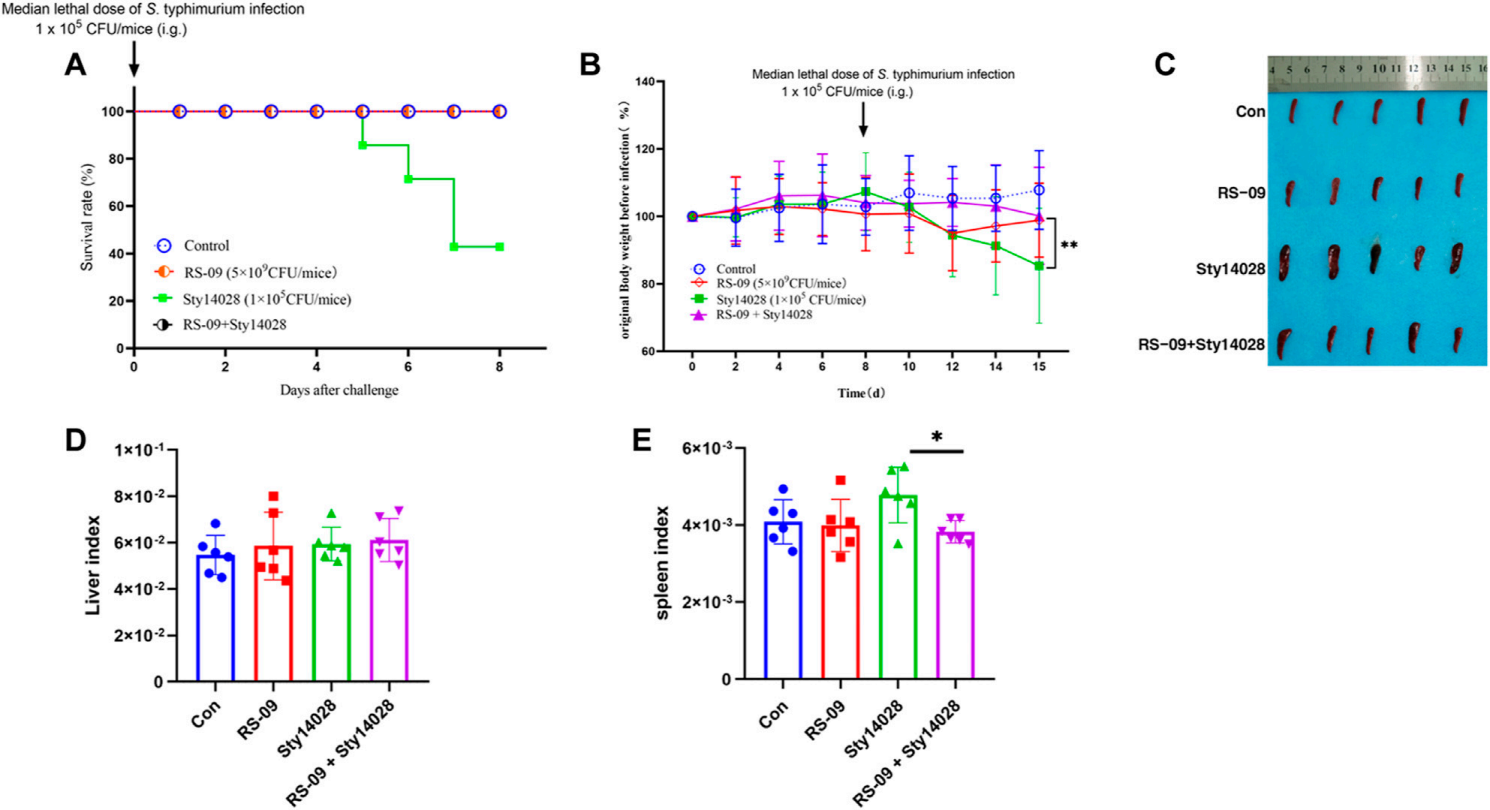
Pretreatment with RS-09 protects mice from *S*. Typhimurium infection. **(A)** Survival rates of mice following RS-09 pretreatment and challenge with pathogenic *S*. Typhimurium. RS-09 was administered to mice for 7 days, and they were then orally injected with a sublethal dose of Sty14028 suspension (10^5^ CFU/mouse). The survival of mice was recorded daily. **(B)** Comparison of body weight loss (current body weight/original body weight before infection) over 8 days post-infection daily. **(C)** RS-09 relieved Sty14028-induced splenomegaly (the scale is shown in the diagram). **(D)** The liver weight index (liver weight/body weight) **(E)** The splenic weight index (spleen weight/body weight). Data are the means ± SD (*n* = 6). *p < 0.05.

Pre-treatment of mice with *L. plantarum* RS-09 was shown to improve the protective efficacy after *S*. typhimurium infection. Mice were treated with a dose of RS-09 (5 × 10^9^ CFU/mouse) for 7 days prior to infection. Then, mice were infected with one dose of *S*. typhimurium 14,028 (Sty14028, 105CFU/mouse) in the presence of RS-09. Figure 2A shows that all of the mice survived RS-09 treatment, whereas 40% of the mice survived after only oral administration of 1 × 10^5^ CFU of Sty14028 (*p* < 0.05). None of mice pretreated with RS- 09 experienced body weight loss, and all survived Sty14028 infection, indicating that protection by RS-09 treatment was significantly improved (*p* < 0.05) (Figure 2B). In contrast, the spleen size and the ratio of the spleen weight to body weight of Sty14028-infected mice exhibited more severe splenic hypertrophy than the mice pre-treated with RS-09 (*p* < 0.05) (Figures 2C,E). Moreover, compared with the control group, the liver/body weight ratio for infected mice showed no significant increase (Figure 2D).

### *L. plantarum* RS-09 Reduces Enteric Pathogenic Bacteria After *S*. Typhimurium Infection

**FIGURE 3:**
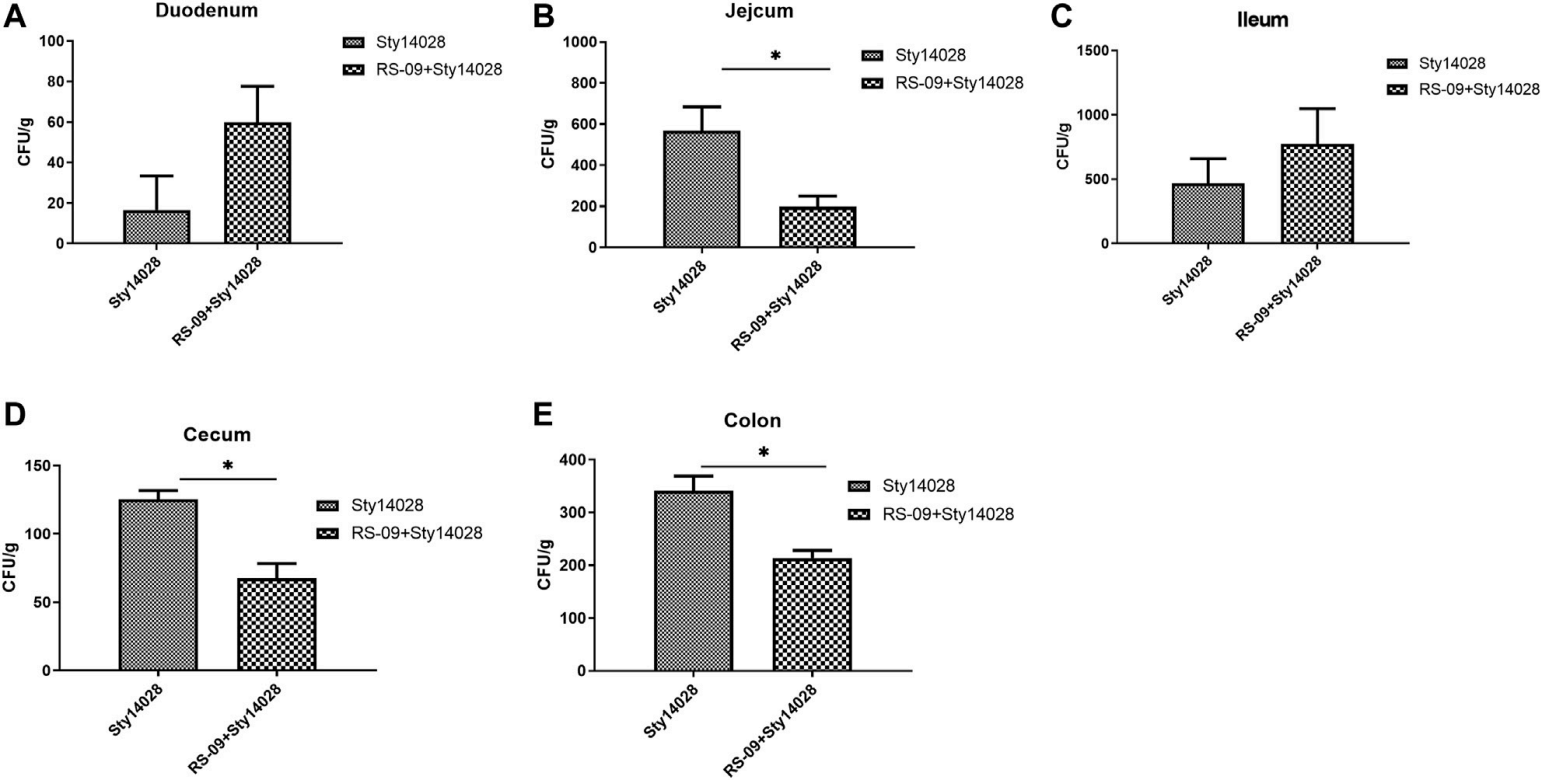
Effect of RS-09 on bacterial load during infection with *S*. Typhimurium. At the indicated times postinfection, dilutions were plated onto XLT-4 agar plates. **(A–E)** Sty14028 loads in the duodenum, jejunum, ileum, caecum and colon for the Sty14028 and RS-09 + Sty14028 groups. The bactericidal activity is shown as the surviving bacterial numbers. Data are the means ± SD (*n* = 6). *p < 0.05.

Previous works proved that *L. plantarum* RS-09 has antibacterial activity on *S.* Typhimurium obviously by utilizing oxford cup ( Supplementary Figure 3S). We next evaluated whether RS-09 in hosts can prevent *S*. Typhimurium colonization in the intestine of mice. The intestinal contents of different intestinal sections from each group were placed on XLT-4 agar plates. Sty14028 loads in the intestine (*p* < 0.05) were lower in the group pre-treated with RS-09 than the Sty14028 group, except in the duodenum and ileum (Figure 3). Furthermore, we did not observe Sty14028 in the intestine of the control or RS-09 groups. Our data suggest that pre-administration of RS-09 can restrain the growth of *S*. Typhimurium *in vivo*.

### Effects of *L. plantarum* RS-09 on Haematological Parameters

**FIGURE 4:**
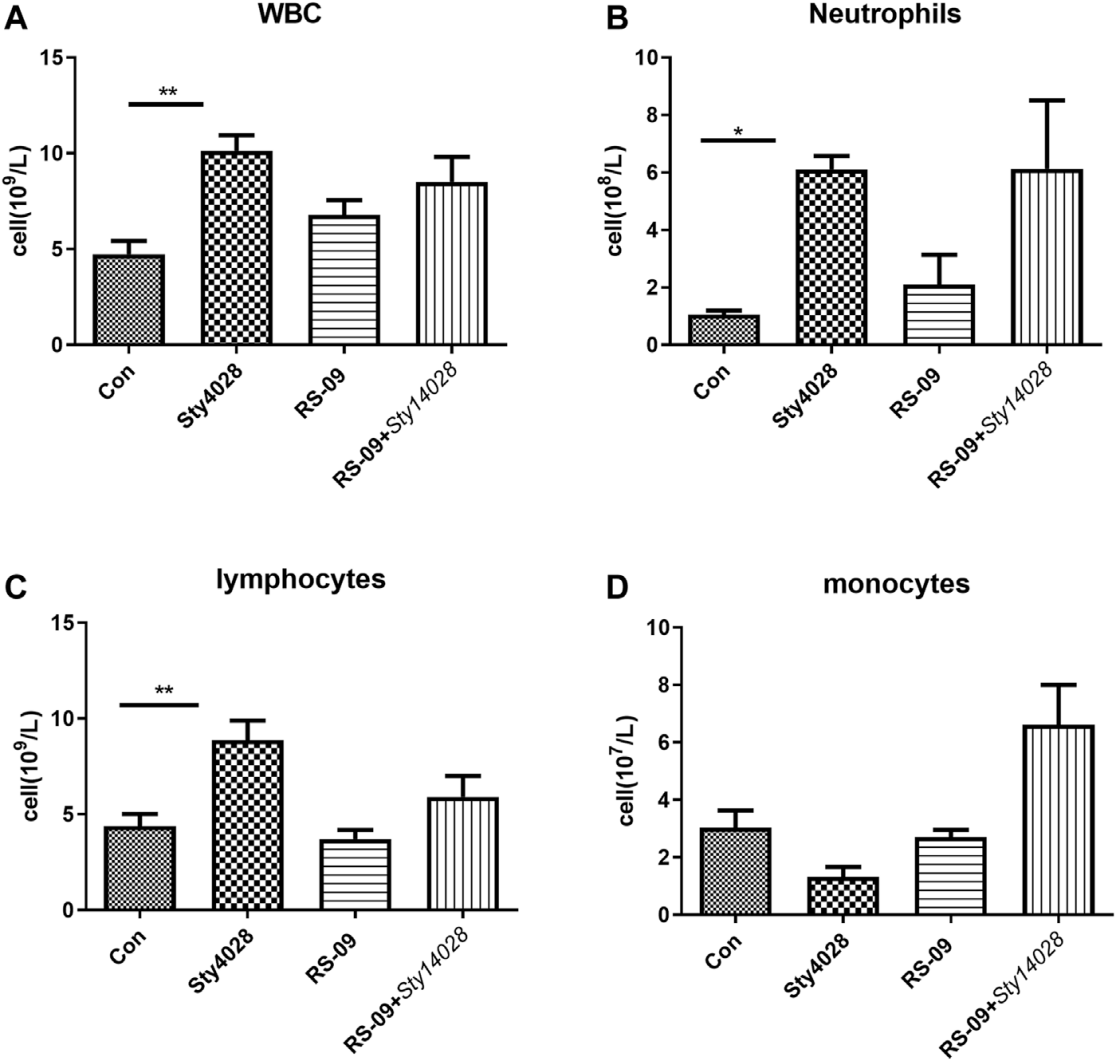
Haematological analysis of mice following an intragastric challenge with *S*. Typhimurium. The total numbers of WBCs **(A)**, as well as the levels of circulating neutrophils **(B)**, lymphocytes **(C)**, and monocytes **(D)**, were evaluated in different groups. Data are the means ± SD (*n* = 3). *p < 0.05; **p < 0.01.

Given the demonstrated *in vivo* effect of bacteria on immune cells, we measured haematological parameters during murine infection with *S*. Typhimurium. At the end of the experiment, animals were sacrificed, and blood was collected for haematological analysis, the results of which are shown in Figure 4. Our findings demonstrated no significant changes in the cellular constituents of blood between the control and RS-09 alone treatment groups, including total white blood cell (WBC), polymorphonuclear neutrophil (PMN), lymphocyte and monocyte counts (Figures 4A,C,D). Following infection, the Sty14028 mice had significantly higher absolute counts, especially WBC (median of 10.13×10^9^ versus 4.73×10^9^ cells/L), neutrophils (median of 6.11×10^8^ versus 1.05×10^8^ cells/L) and lymphocytes (median of 8.87×10^9^ versus 4.38×10^9^ cells/L), compared to control mice blood (Figures 4A–C). In addition, pre-treatment of RS-09 mice showed an increase in monocyte counts throughout infection (Figure 4D). Taken together, these results suggest that the haematological parameters reflect the progression of infection in mice and suggest that pre-treated RS-09 mice are in a recovery phase of *Salmonella* infection compared to Sty14028 mice.

### *L. plantarum* RS-09 Modulates Cytokine Production in *S*. Typhimurium-Infected Mice

**FIGURE 5:**
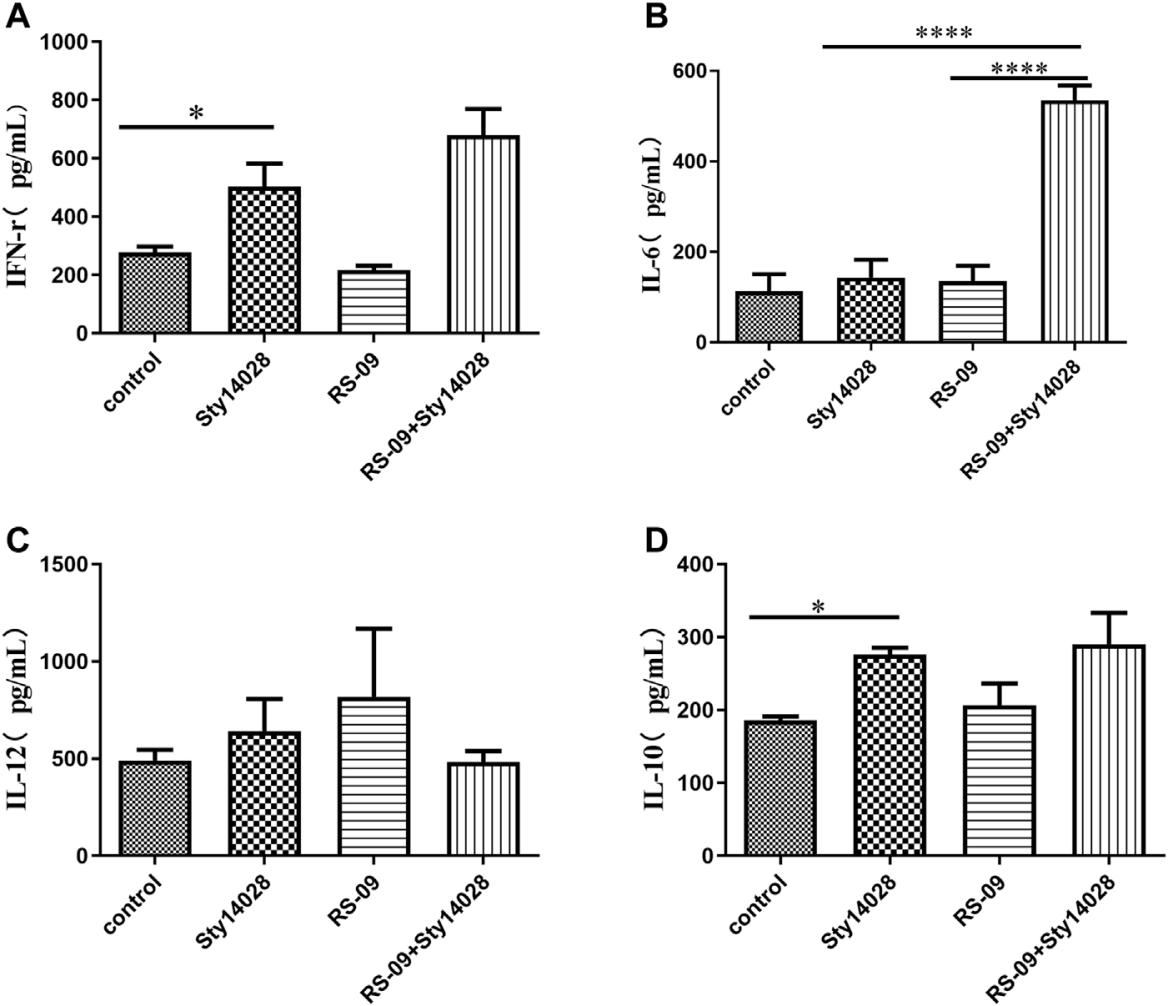
RS-09 modulates cytokine production in mice challenged with pathogenic *S*. Typhimurium. RS-09 (5 × 10^9^ CFU/mouse) was orally administered to mice for 7 days, and they were then orally administered a sublethal dose of Sty14028 suspension (10^5^ CFU/mouse). At 8 d postinfection, serum was collected from the blood to evaluate the expression of **(A)** IFN-γ, **(B)** IL-6, **(C)** IL-12 and **(D)** IL-10. The data are the means ± SD (n = 3). *p* values were calculated by ANOVA. *p < 0.05; ****p < 0.0001.

Inflammatory cytokines are known to be associated with *S.* Typhimurium infection. To better understand the beneficial effects of RS-09 treatment, cytokine levels were measured in the serum of mice by ELISA. The levels of IFN-γ and IL-10 in the serum of infected mice were significantly higher than those in the serum of the PBS mice (*p* < 0.05) (Figures 5A,D). The infected mice fed RS-09 revealed increased levels of IFN-γ and IL-6 compared to *S.* Typhimurium-infected group (Figures 5A,B). However, IL-12 production was not significantly different in the serum of mice across groups (Figure 5C).

### Probiotic RS-09 Promotes M1 Macrophage Polarization and TLR2 Expression

**FIGURE 6:**
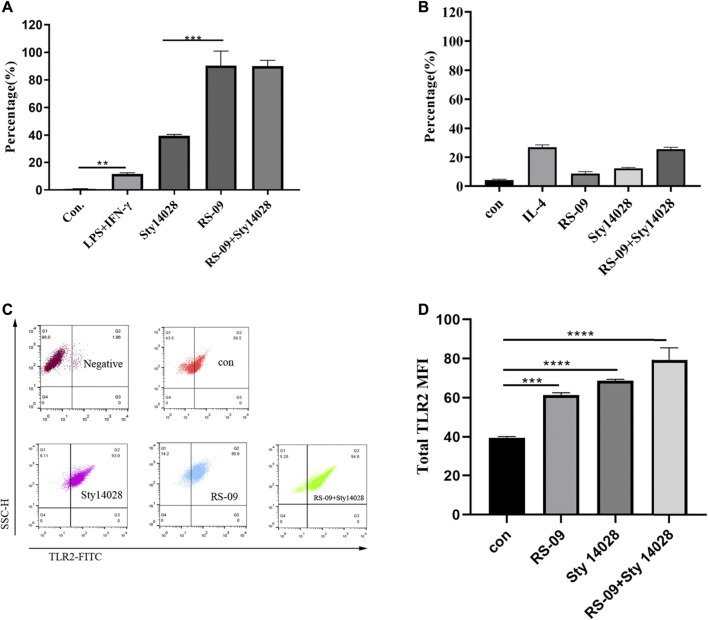
RS-09 stimulates M1 polarization *in vitro*. RAW264.7 macrophages were differentiated and pretreated with RS-09 for 12 h and then treated with Sty14028 for 1 h. **(A)** Representative plot showing the effect of RS-09 on the expression of the M1 marker CD11c^+^CD206^−^ in RAW264.7 macrophages. RAW264.7 cells were treated with LPS (100 ng/ml) plus IFN-γ (20 ng/ml) to differentiate into M1 (M1 positive control) macrophages. **(B)** Detection of M2 macrophage polarization-related markers by flow cytometry. RAW264.7 cells were treated with IL-4 (40 ng/ml) to differentiate into M2 macrophages (M2 positive control). **(C)** Representative plot showing the expression of TLR2 in RAW264.7 macrophages. **(D)** Quantitation of TLR2-positive cells derived from RAW264.7 cells after different combination treatments for 12 h **p < 0.01; ***p < 0.001; ***p < 0.0001. Data are the means ± SD of three independent experiments.

M1 macrophages promote inflammatory responses and clear pathogens (Italiani and Boraschi, 2014). Macrophages treated with Sty14028 showed a tendency toward M1 polarization. Flow cytometry showed that M1 expression in the Sty14028 group was significantly higher than that in the LPS + IFN-γ group (*p* < 0.01), and the M2 expression level was similar to that in the IL-4 group (Figures 6A,B). We subsequently attempted to identify Sty14028-infected macrophage cell subtypes after RS-09 pre-treatment. RS-09 treatment of RAW264.7 cells resulted in increases in CD11c^+^ populations by 114-fold compared with the control (Figure 6A). We also detected the proportion of M1 macrophages in the mesenteric lymph nodes. The results showed RS-09 did induce M1 macrophage polarization compared with control group *in vivo* ( Supplementary Figure 6S). Therefore, increases in M1 macrophage polarization by RS-09 might contribute to improving protection against *Salmonella* infection.

TLRs in the cell membrane of macrophages contribute to the early recognition of pathogen-associated molecular patterns (PAMPs) of external pathogens (Kawai and Akira, 2011). In addition, TLR signalling has been reported to induce the activation of macrophage inflammatory responses to fight external pathogens (Fitzgerald and Kagan, 2020). Thus, we investigated the expression of TLR2 by FCM analysis. As shown in Figure 6C, compared with the control group, both RS-09 and Sty14028 exposure significantly upregulated the expression of TLR2.

### Effect of RS-09 on *S*. Typhimurium-Induced ROS Production in RAW264.7 Cells

**FIGURE 7:**
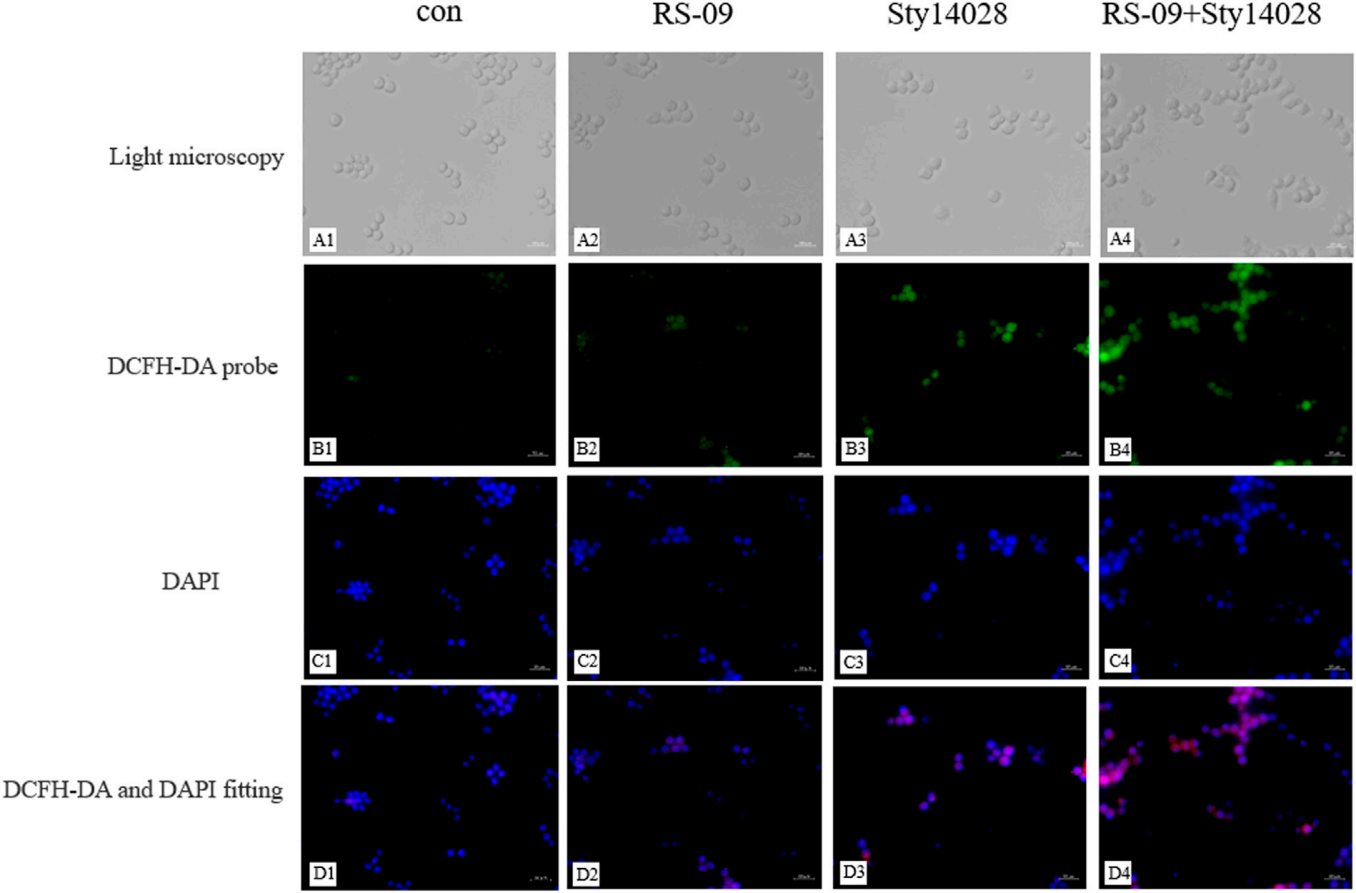
Effect of RS-09 on Sty14028-induced ROS generation in RAW264.7 macrophages. RAW264.7 cells were cultured in DMEM for 12 h and then incubated with the probe DCFH-DA (green) for 30 min. DNA was stained with DAPI (blue) for 10 min **(A1–A4)** The cell morphology was observed by light microscopy. **(B1–B4)** ROS detection was performed using fluorescence macroscopy **(C1–C4)** Treated cells were stained with DAPI and observed under fluorescence microscopy **(D1–D4)** Merged image of DCFH-DA and DAPI. These experiments were repeated independently 3 times (400×).

Macrophages with ROS production can protect against microbial pathogens. With this in mind, we next detected whether pre-treatment with RS-09 was able to induce infected RAW264.7 macrophages to produce ROS by the fluorescent probe DCFH-DA (green) with immunofluorescence microscopic studies. As demonstrated in Figure 7, RAW264.7 cells infected with the Sty14028 strain and stained with DCFH-DA displayed significantly higher levels of fluorescence, whereas Sty14028-induced ROS generation was significantly improved by RS-09 pre-treatment. We also analyzed the quantitative fluorescence of ROS (Supplementary Figure 7S). The ROS levels of pre-treatment with RS-09 cells were significantly higher than that in the Sty14028 group (*p* < 0.0001). This reconfirmed that the RS-09 strain could induce ROS production during resistance to *Salmonella* infection.

### Effect of RS-09 on NO Production by RAW264.7 Cells

**FIGURE 8:**
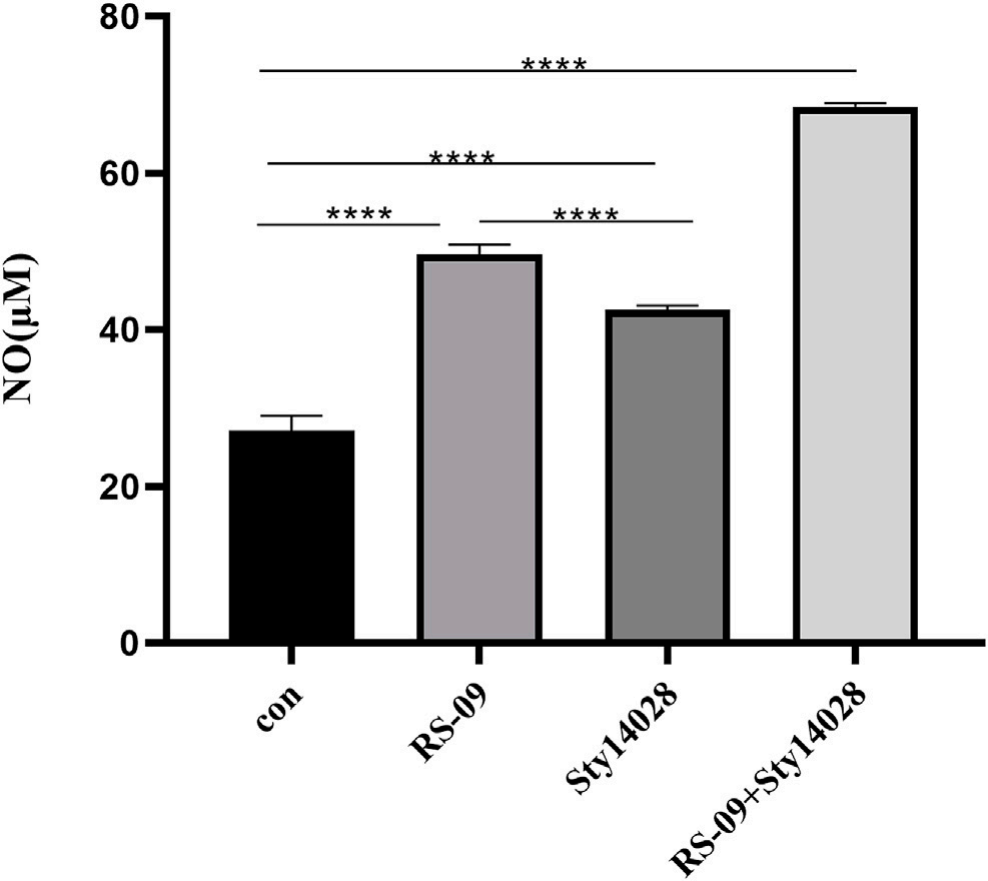
Effect of RS-09 on nitrite (NO) production in bacteria-stimulated RAW264.7 macrophages. The content of NO in the culture supernatant was measured by the Griess reaction assay. The values represent the means ± SD of three independent experiments. ****p < 0.0001.

NO is an important biological effector molecule and the main catabolite in M1 macrophages, which has cytotoxic effects on invading pathogenic microorganisms (Li et al., 2015). To investigate the ability of RS-09 to produce NO in RAW264.7 macrophages, we examined the NO levels in the culture supernatants of cells stimulated with RS-09 (4 × 10^7^ CFU) for 12 h and those stimulated with Sty14028 (4 × 10^7^ CFU) for 1 h. As shown in Figure 8, compared with the control group, Sty14028 significantly stimulated NO production in RAW264.7 cells (*p* < 0.0001). RS-09 also induced NO production in RAW264.7 cells. However, after pretreatment with RS-09, the NO levels of infected cells increased to 49.65 μM, which was significantly higher than that in the Sty14028 group (*p* < 0.0001). This increased production of NO suggests that RS-09 may activate the bactericidal activities of macrophages and potentiate the M1 polarity of RAW264.7 macrophages.

### RS-09 Modulates TLR2/NF-κB Signalling to Clear *S*. Typhimurium in M1 Macrophages

**FIGURE 9:**
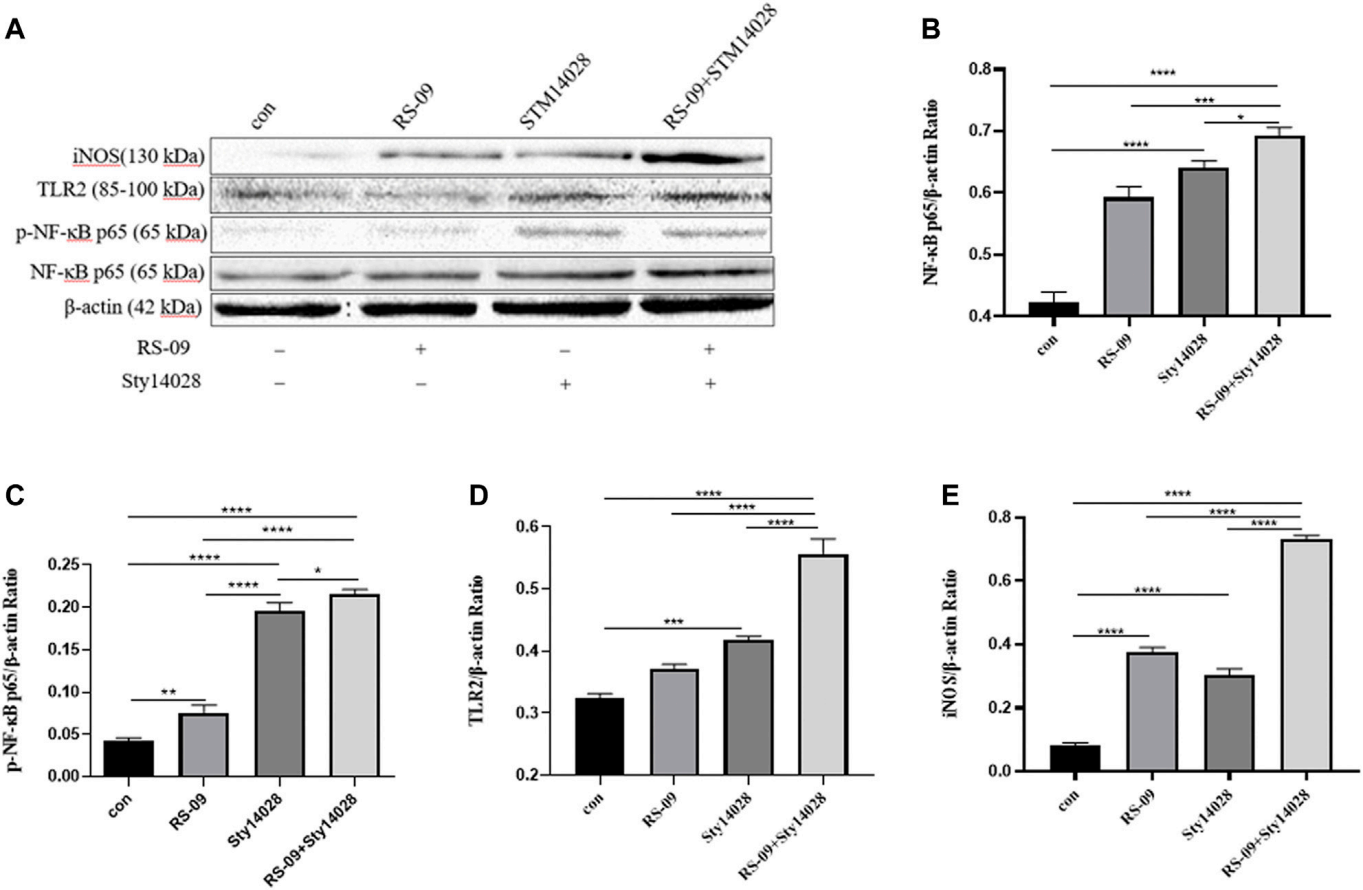
RS-09 promotes the expression of NF-κB in M1-type macrophages. Raw264.7 cells were stimulated with RS-09 alone, Sty14028 alone, or both RS-09 and Sty14028 for 12 h. **(A)** Levels of iNOS, TLR2, p-NF-κB and NF-κB were detected using Western blotting **(B–D)** The quantified values of NF-κB, p-NF-κB, TLR2 and iNOS **(E)** expression in RAW264.7 cells were determined during 12 h of incubation. The image grey value was analysed using AlphaEaseFC software (Alpha Innotech, CA, United States). All values are expressed as the means ± S.D (*n* = 3). *p < 0.05; **p < 0.01; ***p < 0.001; ****p < 0.0001.

NF-κB is a key member of the signalling pathway responsible for the activation of inflammatory cytokine secretion by monocytes-macrophages. It has also been reported that nuclear factor-κB (NF-κB) activated by the stimulation of TLRs is an essential master regulatory signalling pathway that contributes to the production of immunomodulators by macrophages and other innate immune cells against external pathogens (Kawai and Akira, 2007; Dorrington and Fraser, 2019). TLRs, among PRRs of macrophages, contribute to the early recognition of external pathogens (Kawai and Akira, 2011), and TLR signalling has been reported to induce the secretion of various immunomodulators (Strieter et al., 2003). To elucidate the molecular mechanism of RS-09-induced M1 polarization *in vitro*, we analysed the TLR2/NF-κB pathway by immunoblot analysis. We found that the iNOS level of M1 macrophage-related markers increased after treatment with RS-09 and Sty14028 (Figure 9A). Analysis of intracellular proteins showed that the group infected with Sty14028 exhibited higher levels of NF-κB expression than the control group (Figure 9B) (*p* < 0.001). Notably, stimulation of RS-09-treated Raw264.7 cells combined with Sty14028 significantly increased NF-κB levels (*p* < 0.001). However, concurrent treatment with RS-09 and Sty14028 resulted in higher NF-κB levels than treatment with RS-09 or Sty14028 alone (*p* < 0.001; *p* < 0.05). In line with the change in the level of NF-κB expression, the protein levels of iNOS and TLR2 in RAW246.7 macrophages were also upregulated after RS-09 treatment. Based on these results, RS-09 can promote the inflammatory response of Sty14028-infected M1 macrophages through the TLR2-mediated NF-κB signalling pathway.

## Discussion

*Salmonella-*mediated inflammation is dependent on an intricate series of host-pathogen interactions affected by the immunologic status of the host. In this study, we evaluated the protective capacity of the immune response by *L. plantarum* RS-09 against a challenge with *S*. Typhimurium. The findings indicated that RS-09 leads to anti-inflammatory effects by reducing *S*. Typhimurium colonization in the intestine, upregulating the expression of proinflammatory cytokines and ameliorating the oxidative stress response.

*S*. Typhimurium is first observed in intestinal tissues at the beginning of infection and then crosses the lining of intestinal epithelial cells, translocates to extraintestinal organs such as the spleen and liver, and survives in host immune cells (such as macrophages and dendritic cells). As such, colonization by *S*. Typhimurium in different intestinal tissues was inconsistent in the present study. The number of *S*. Typhimurium duodenal and ileac colonies in the RS-09 + Sty14028 group was higher than in the Sty14028 group. The *S*. Typhimurium colonies in other intestinal sections after RS-09 treatment were reduced compared with the Sty14028 group. Brewer reported less *Salmonella enterica* colonization in the ileum of probiotic-supplemented calves than control calves (Brewer et al., 2014). According to Finlay and Brumell (Finlay and Brumell, 2000), the effect of probiotics on the colonization of *S*. Typhimurium in gastrointestinal tracts is highly dependent on adhesion and persistence (Duary et al., 2011). In our previous investigation, we evaluated probiotic strains of *L. plantarum* RS-09 for their ability to adhere to HT-29 cells. On comparative evaluation based on adhesion score, *L. plantarum* RS-09 was found to be the most adhesive strain and could therefore inhibit *S*. Typhimurium adhesion to HT-29 cells. Similarly, other workers have also reported the competitive exclusion of pathogens by lactobacilli (Collado et al., 2007; Pham et al., 2009; Zhang et al., 2009). In our results, the competitive profile of *S*. Typhimurium by *L. plantarum* RS-09 strains was different in the gut, indicating that the pathogen inhibition capacity of probiotic strains was possibly due to different intestinal section surface characteristics. This suggests that the mechanism involved in inhibition is complicated and that many factors may be involved.

Infection with *S*. Typhimurium first induces local inflammatory responses in the intestinal mucosa. Additionally, severe infections can cause systemic inflammation, which can influence other organs. A previous study illustrated that rats supplemented with *Lactobacillus casei* had no difference in either liver or spleen weight compared with control rats (De Waard et al., 2002). In agreement, our study in mice demonstrated no difference in either liver index or spleen index between the *L. plantarum* RS-09 group and a control group. Moreover, Jain et al. demonstrated no difference in liver weight between a *Lactobacillus casei* group and a control group after a *Salmonella* Typhimurium challenge (Jain et al., 2008). This result was consistent with our findings. In addition, the spleen index tended to decrease in the RS-09 + Sty14028 group compared with the Sty14028 group, the difference was significant (*p* < 0.05).

Additionally, we evaluated the biochemical parameters of mice challenged with *S*. typhimurium and supplemented with *L. plantarum* RS-09 inoculum. Significant differences were observed in total WBC, neutrophil and lymphocyte counts after treatment with the *S*. Typhimurium inoculum. We detected no difference in total monocyte counts between the control and RS-09 groups. Furthermore, the RS-09 + Sty14028 group had an increased Polymorphonuclear Neutrophils (PMN) percentage compared with the Sty14028 group. As reported earlier, an increase in PMN count is one of the first innate immune actions observed in response to *Salmonella* infection (Wick, 2004). Furthermore, probiotics have been demonstrated to increase the production of immune cell populations, including neutrophils and monocytes (Liang et al., 2020). While there was no significant increase in WBC or lymphocyte concentrations in the RS-09 group compared with the control group, the concentrations were on average reduced in the RS-09 + Sty14028 treatment group compared to Sty14028 mice. Alternatively, the attenuated response of WBCs and lymphocytes after RS-09 + Sty14028 treatment may also indicate that the infection was better controlled locally or by some other component of the immune system.

*Salmonella* is an intracellular pathogen that elicits an inflammatory response once taken up by intestinal epithelial cells. This action can activate innate immune cells, one pathway being through the bacterial component recognized by TLRs, and results in the release of cytokines, such as IFN-γ, into circulation (Benoun et al., 2018; Gabr et al., 2021). M1 Macrophages can be stimulated by IFN-γ after intracellular pathogen challenge (Ramarathinam et al., 1993). M1 macrophages are pro-inflammatory and produce pro-inflammatory cytokines such as IFN-γ, IL-6 and IL-12, which destroying pathogens (Shapouri-Moghaddam et al., 2018). Similar results were observed in our study, where the IFN-γ level increased in *Salmonella* Typhimurium-challenged mice. Additionally, the monocyte ratio of RS-09 + Sty14028 mice was significantly increased compared with that of any other group. This observation was supported by Shida et al. (Shida et al., 2006), who observed that a *Lactobacillus casei* strain could be phagocytosed by human mononuclear cells and then induce the production of IFN-γ and IL-12. Elevation of serum IL-12 levels in the RS-09 group was consistent with our findings. The lower expression of IL-12 in the RS-09 + Sty14028 group may be related to the process of inflammatory reaction. With the inflammation continues, it can cause tissue damage. Therefore, RS-09 secrete amounts of IL-10 to suppress the inflammation, contribute to tissue repair, remodeling and retain homeostasis. This observation was supported by Duan, B (Duan et al., 2021), who observed that *Lactobacillus rhamnosus* GG strain induced the production of IL-10. When the inflammation improved, it maybe associated with the decreased the expression of Inflammatory cytokine IL-12 in the RS-09 + Sty14028 group.

Accumulated evidence has shown that macrophages can be phenotypically polarized by microbes into two main groups: M1 macrophages and M2 macrophages. The type of polarized phenotype depends on many factors, such as bacterial strain, dose and exposure time (Yang Wang et al., 2020). The M1 phenotype favours vital microbicidal properties. Our current work demonstrated that probiotic RS-09 influences the outcome of *S*. Typhimurium infection and is associated with M1 macrophage polarization (Martinez et al., 2008). We have shown that the presence of RS-09 promotes the development of a robust proinflammatory response in mice, characterized by the expression of proinflammatory cytokines, ROS, and NO for clearance of the bacteria (Liu et al., 2014). In our study, we found that RS-09 pre-treatment significantly increased the expression of proinflammatory cytokines (IL-6), suggesting that RS-09 may serve as a regulator to improve the proinflammatory response of macrophages. In previous studies, an established probiotic cocktail (LDB-ST) was reported to elevate the expression of proinflammatory macrophage markers (IL-6, IL-1β, IL-12, and TNF-α) in RAW264.7 cells (Guha et al., 2019). As mentioned in previous studies, ROS production has been recognized as one of the earliest host innate immune responses followinng microbial invasion (Li et al., 2019). In agreement with these findings, we found ROS production in RAW264.7 cells in response to RS-09 treatment. NO has been identified as one of the major effector molecules produced by activated macrophages and is the main catabolite of iNOS in M1 macrophages (Kim et al., 2006; Wang et al., 2008; Chiou et al., 2011). Our study demonstrated that RS-09 promotes macrophage antimicrobial activity against *S*. Typhimurium, which is also consistent with M1 macrophage function.

The inflammatory response is regulated by complex and cross-linked endogenous cellular signalling pathways and their modulators (Takeuchi and Akira, 2010). Evidence has shown that host–microbe interactions depend on pattern recognition receptors; for instance, TLR2 can recognize many different microbial components (Beutler et al., 2006; Takeuchi and Akira, 2010). Many pathways and transcription factors are involved in macrophage polarization, such as nuclear factor NF-κB (Liu et al., 2014). There is evidence that NF-κB signalling pathways are involved in macrophage polarization (Liu et al., 2017; Luo et al., 2020). M1 phenotypic macrophages can be activated by bacterial components via the TLR2/NF-κB signalling pathway. The main role of the TLR2/NF-κB pathway is to induce the expression of inflammatory cytokines (such as iNOS), which are biomarkers of M1 macrophages (Cunha et al., 2016). In the present study, Western blot results showed that RS-09 increased the expression of TLR2, NF-κB and iNOS in RAW264.7 cells. Further studies are needed to define the role of probiotic component interactions with TLR2 receptors and signalling events.

## Conclusion

In conclusion, our data demonstrate that probiotic RS-09 induces M1 polarization of macrophages and has potent antimicrobial activity against *S*. Typhimurium by activating TLR2/NF-κB signalling pathways. These findings are beneficial for a better understanding of the mechanism by which RS-09 modulates the host immune response against pathogen infection, especially the molecular mechanisms of probiotics in regulating M1 polarization.

## Data Availability

The original contributions presented in the study are included in the article/Supplementary Material, further inquiries can be directed to the corresponding authors.
