# Erratum: *Lactobacillus plantarum* RS-09 Induces M1-Type Macrophage Immunity Against *Salmonella typhimurium* Challenge *via* the TLR2/NF-κB Signalling Pathway

**DOI:** 10.3389/fphar.2022.937618

**Published:** 2022-06-03

**Authors:** Frontiers Production Office

**Affiliations:** Frontiers Media SA, Lausanne, Switzerland

**Keywords:** *Lactobacillus plantarum*, *Salmonella typhimurium*, macrophage, macrophage polarization, M1 macrophage, TLR2

Due to a production error, the formatting of ten values and two words (“mL” and “Interleukin-6”) was incorrect. Corrections have been made to the **Materials and Methods** section:

**Bacterial Infection Assay in Mice:**

“**Figure 1** shows the experimental design. Twenty-four mice were randomly divided into four groups (six mice per group): normal control group treated with PBS alone, control group treated with Sty14028 alone, one group treated with *L. plantarum* RS-09, and one group treated with *L. plantarum* RS-09 with *S. typhimurium* 14,028. The RS-09 group and RS-09 with Sty14028 group received an intragastric administration of 200 μL of a suspension containing 5 × 10^9^ CFU/mouse of live RS-09 once daily for 7 consecutive days *via* a feeding needle. On the 8th day of RS-09 in the Sty14028 group, mice were infected with a 200 μL median lethal dose of *S. typhimurium* 14,028 (1 × 10^5^ CFU/mouse). All animal weights and survival rates were monitored daily, and mice were euthanized 8 days post-challenge.”

**Blood Sampling:**

“For each mouse, 1.0 mL of blood from the caudal vein was collected. The blood samples were divided into two parts. One was treated using sodium citrate to obtain anticoagulated whole blood to detect routine histopathology by an automatic blood cell analyser (Rayto RT-7600S, Shenzhen, China), and the other was stored at 4°C for 4 h to collect serum for cytokine measurements.”

**Cytokine Quantification:**

“Cytokines, including mouse IFN-γ, IL-6, and Interleukin-12 (IL-12), were quantified in serum using enzyme-linked immunosorbant assays (ELISAs) following the manufacturer’s protocols (R&D Systems, MN, United States). ELISA for IL-10 (Cat. no. 430603) using serum was also performed following the manufacturer’s protocols (BioLegend, CA, United States). All ELISAs were performed using 96-well high-binding ELISA plates in ELISA kits, and plates were read at a wavelength of 450 nm using a Synergy HTTR microplate reader (Bio-Tek Instrumentation, VT, United States).”

**Macrophage Stimulation *In Vitro*:**

“For macrophage polarization assays, RS-09 and Sty14028 were collected by centrifugation and washed three times with PBS. RAW264.7 cells were seeded into a 24-well culture plate at a concentration of 2 × 106 per well. RS-09 was then applied to infect macrophages at an MOI of 20:1 (4 × 10^7^ CFU: 2 × 106 RAW264.7) for 12 h (*n* = 3 for each group). RAW264.7 cells were then washed with PBS twice. Sty14028 was then applied to infect the macrophages at an MOI of 20:1 (4 × 10^7^ CFU: 2 × 106 RAW264.7). After incubation for 1 h, the cells were washed three times and treated with 200 μL of gentamicin sulfate (100 μg/ml) for 1 h. RAW264.7 cells were then washed with PBS twice and incubated for 12 h with DMEM (including 20 μg/ml of gentamicin sulfate). The cells were harvested for the detection of macrophage phenotype, reactive oxygen species (ROS), and proteins with altered expression, and culture supernatants were simultaneously assayed for NO.”

**Western Blot:**

“After treatment with bacteria, cells were washed with ice-cold PBS and lysed in RIPA lysis buffer containing 1 mM PMSF. With centrifugation at 12,000 rpm and 4°C for 20 min, the protein concentration was determined by the BCA protein quantitative method (Thermo, MA, United States). Equal amounts of protein (50 μg per lane) were separated by 12% sodium dodecyl sulfate–polyacrylamide gel electrophoresis (SDS–PAGE) and transferred to nitrocellulose membranes on ice. The membrane was blocked with 5% skimmed milk (Cell Signalling Technology, MA, United States) for 2 h at room temperature and incubated with iNOS (1:1,000, Cell Signalling Technology, MA, United States).”

Corrections have been made to the **Results** section:

**Effects of Pretreatment of Mice With *L. plantarum* RS-09 on *S. typhimurium* Infection:**

“Pre-treatment of mice with *L. plantarum* RS-09 was shown to improve the protective efficacy after *S. typhimurium* infection. Mice were treated with a dose of RS-09 (5 × 10^9^ CFU/mouse) for 7 days prior to infection. Then, mice were infected with one dose of *S. typhimurium* 14,028 (Sty14028, 105CFU/mouse) in the presence of RS-09. **Figure 2A** shows that all of the mice survived RS-09 treatment, whereas 40% of the mice survived after only oral administration of 1 × 10^5^ CFU of Sty14028 (*p* < 0.05). None of mice pretreated with RS- 09 experienced body weight loss, and all survived Sty14028 infection, indicating that protection by RS-09 treatment was significantly improved (*p* < 0.05) (**Figure 2B**). In contrast, the spleen size and the ratio of the spleen weight to body weight of Sty14028-infected mice exhibited more severe splenic hypertrophy than the mice pretreated with RS-09 (*p* < 0.05) (**Figures 2C,E**). Moreover, compared with the control group, the liver/body weight ratio for infected mice showed no significant increase (**Figure 2D**).”

**Effect of RS-09 on NO Production by RAW264.7 Cells:**

“NO is an important biological effector molecule and the main catabolite in M1 macrophages, which has cytotoxic effects on invading pathogenic microorganisms (**Li et al., 2015**). To investigate the ability of RS-09 to produce NO in RAW264.7 macrophages, we examined the NO levels in the culture supernatants of cells stimulated with RS-09 (4 × 10^7^ CFU) for 12 h and those stimulated with Sty14028 (4 × 10^7^ CFU) for 1 h. As shown in **Figure 8**, compared with the control group, Sty14028 significantly stimulated NO production in RAW264.7 cells (*p* < 0.0001). RS-09 also induced NO production in RAW264.7 cells. However, after pre-treatment with RS-09, the NO levels of infected cells increased to 49.65 μM, which was significantly higher than that in the Sty14028 group (*p* < 0.0001). This increased production of NO suggests that RS-09 may activate the bactericidal activities of macrophages and potentiate the M1 polarity of RAW264.7 macrophages.”

The publisher apologizes for this mistake. The original version of this article has been updated.

