# Supplementary material for: Lactobacillus plantarum RS-09 Induces M1-Type Macrophage Immunity Against Salmonella Typhimurium Challenge via the TLR2/NF-κB Signalling Pathway
Source: Front Pharmacol. 2022 Mar 7;13:832245. doi: 10.3389/fphar.2022.832245 (PMC8959098; doi:10.3389/fphar.2022.832245)
Supplement: Supplementary file 1 [file Presentation1.pptx]

## Slide 1
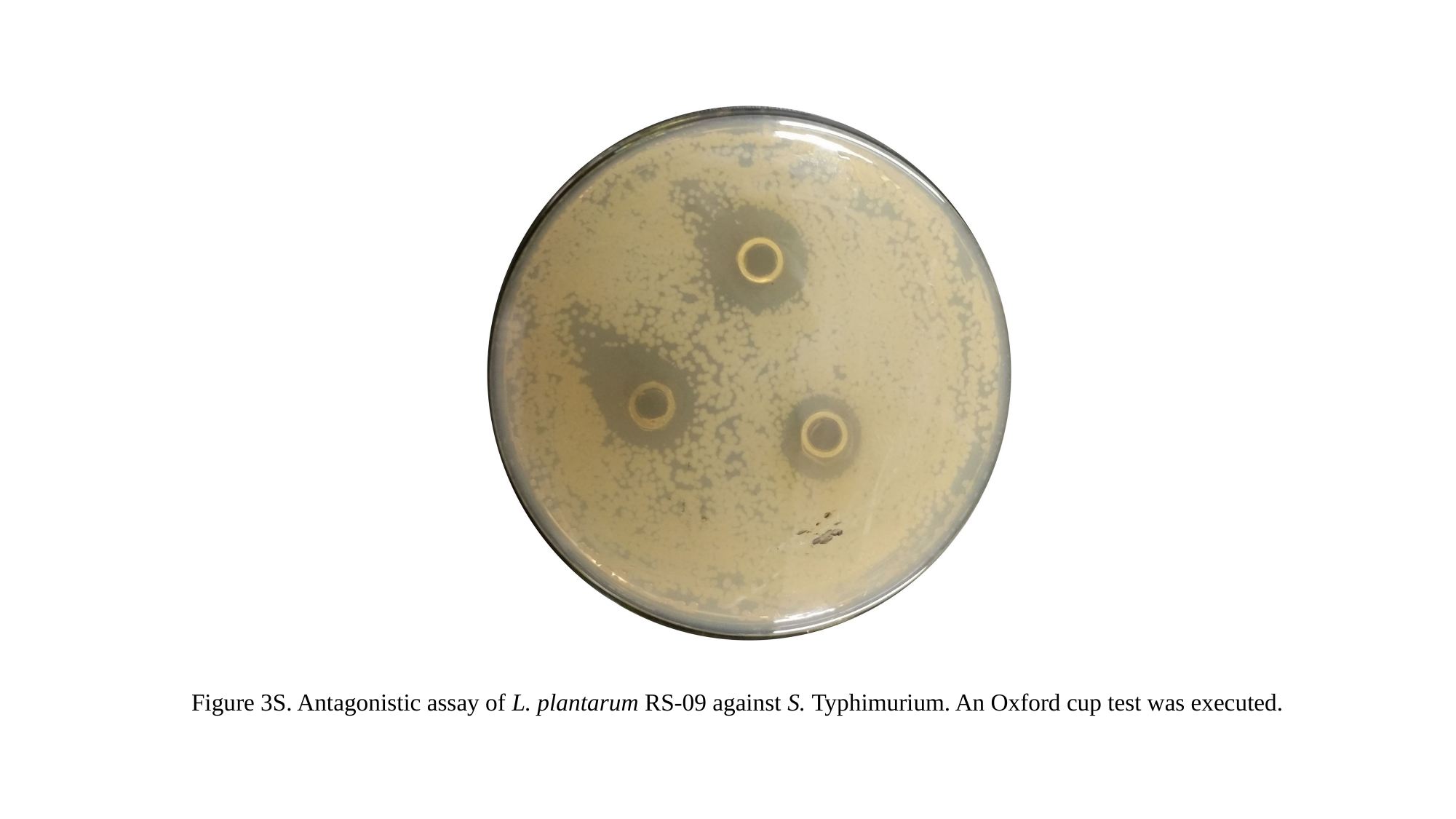

Figure 3S. Antagonistic assay of L. plantarum RS-09 against S. Typhimurium. An Oxford cup test was executed.

## Slide 2
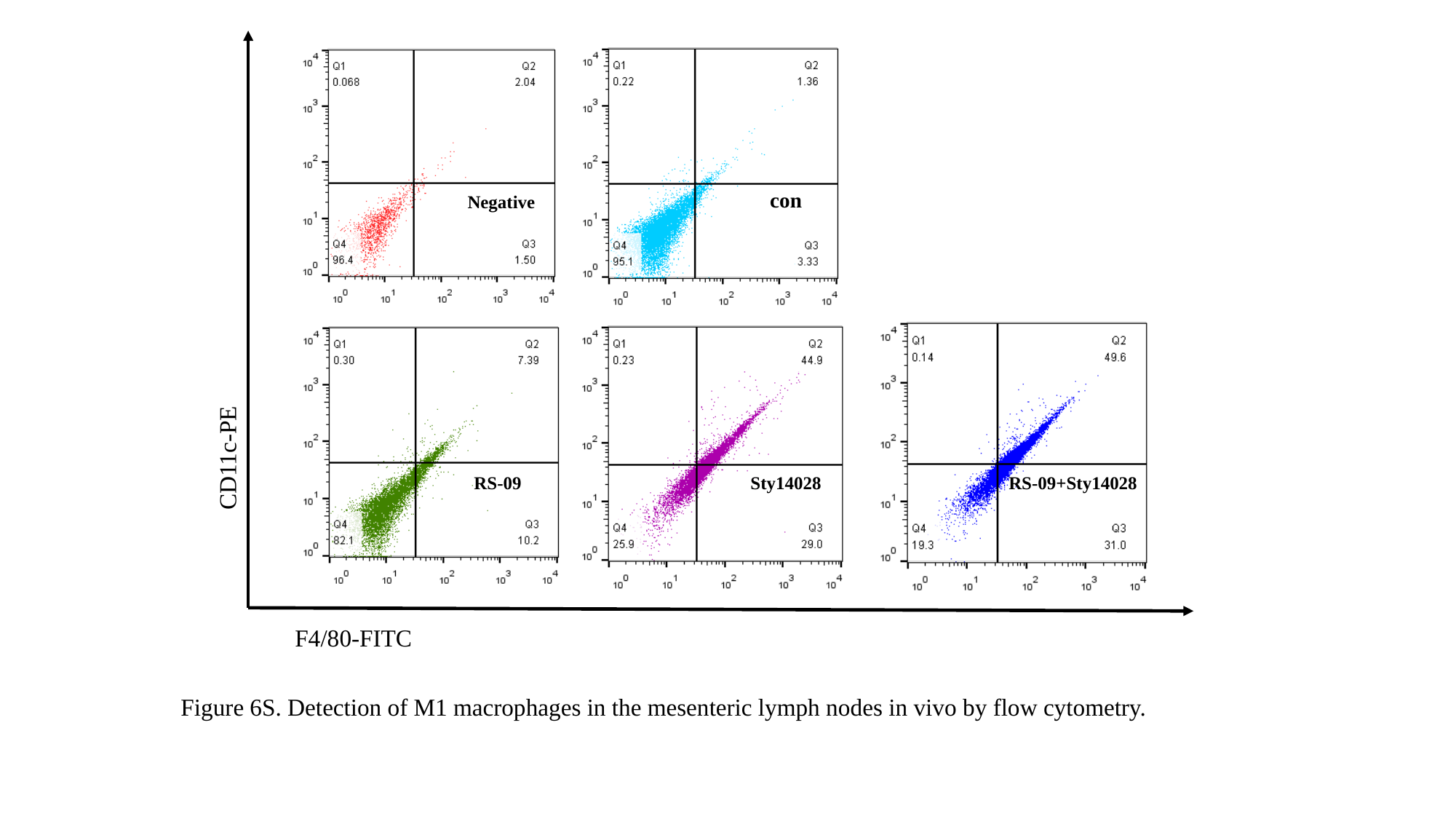

con
Negative
CD11c-PE
Sty14028
RS-09+Sty14028
RS-09
F4/80-FITC
Figure 6S. Detection of M1 macrophages in the mesenteric lymph nodes in vivo by flow cytometry.

## Slide 3
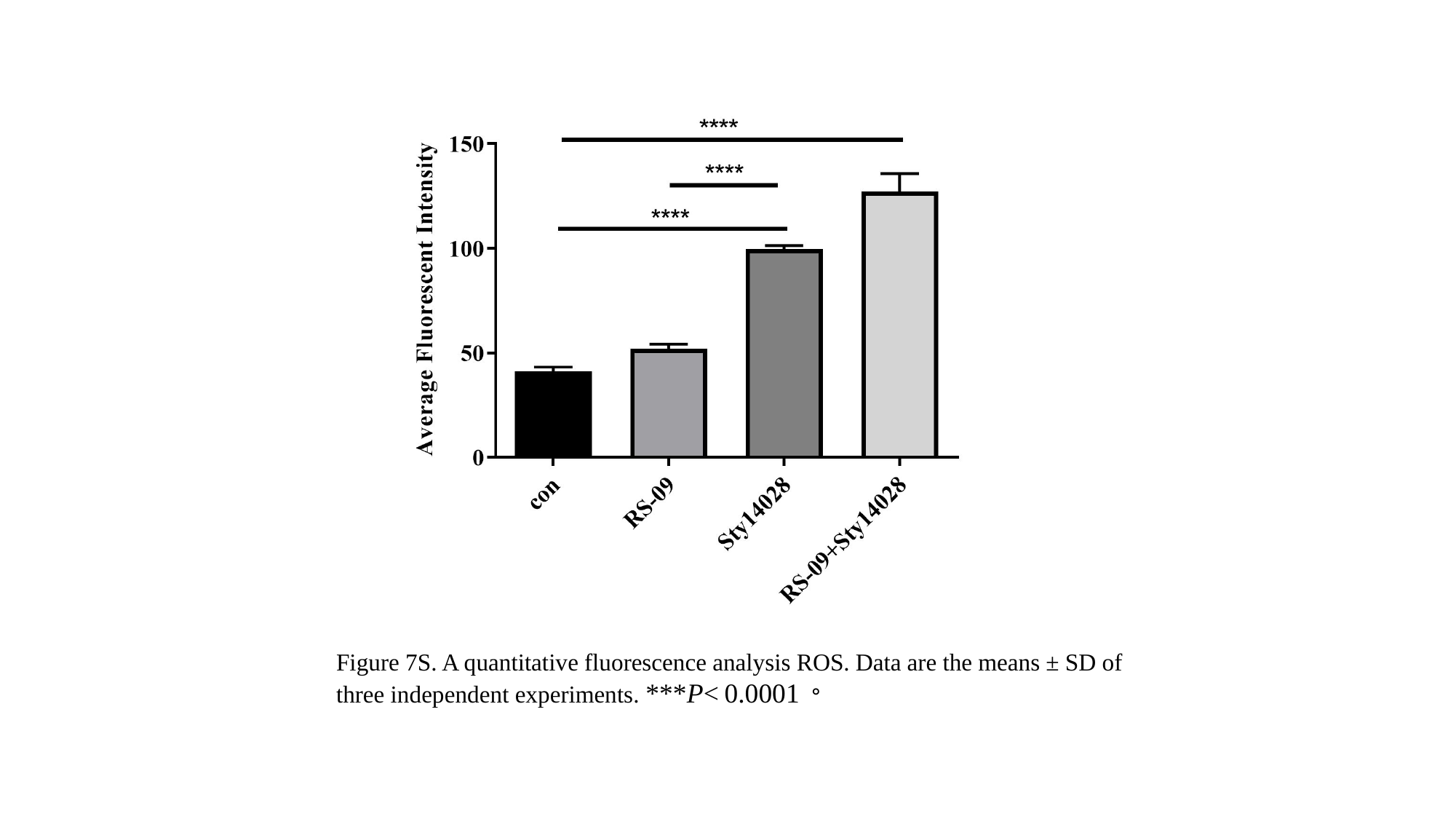

Figure 7S. A quantitative fluorescence analysis ROS. Data are the means ± SD of three independent experiments. ***P< 0.0001。
